# AI-enabled RF data synthesis for breast ultrasound: efficacy in quantitative ultrasound tissue characterization

**DOI:** 10.1038/s41598-026-43319-9

**Published:** 2026-03-07

**Authors:** Nasrin Sheibani-Asl, Laurentius O. Osapoetra, Gregory J. Czarnota, Ali Sadeghi-Naini

**Affiliations:** 1 Department of Electrical Engineering and Computer Science, Lassonde School of Engineering, York University, Toronto, ON Canada; 2 Department of Radiation Oncology, Odette Cancer Centre, Sunnybrook Health Sciences Centre, Toronto, ON Canada; 3 Physical Sciences Platform, Sunnybrook Research Institute, Sunnybrook Health Sciences Centre, Toronto, ON Canada; 4 Department of Medical Biophysics, University of Toronto, Toronto, ON Canada

**Keywords:** Generative AI, Quantitative Ultrasound, B-mode Ultrasound Imaging, Conditional Generative Adversarial Networks (cGANs), Vision Transformer (ViT), Synthetic Radio-Frequency (RF) Data, Breast Lesion Characterization, Cancer, Computational biology and bioinformatics, Medical research

## Abstract

Quantitative ultrasound (QUS) methods can derive insightful biomarkers from raw radiofrequency (RF) signals for tissue characterization and monitoring, but their clinical adoption is limited by the inaccessibility and storage burden of RF data. This study is the first to investigate the potential of deep generative models in synthesizing RF data from standard B-mode images and evaluate their efficacy in downstream QUS analysis. Three conditional generative adversarial networks (cGAN), namely Pix2Pix, a shallow ViT‐based cGAN, and a deep ViT‐based cGAN, were adapted and trained on a large paired dataset of RF/B‐mode frames (21,174 training, 3,456 validation, 8,919 test frames) collected from 152 patients (98 patients in the training, 16 in validation, and 38 in the test set) with suspicious breast lesions. The synthesized RF data were assessed using sample-level evaluation metrics, and via a benign-malignant lesion classification task based on the corresponding QUS features. The generative models achieved a structural similarity index measure (SSIM) of 0.82 ± 0.05 on the synthetic RF data and an average peak signal-to-noise ratio (PSNR) of about 33 dB on the corresponding B-mode images, confirming strong reconstruction fidelity. In the lesion classification experiments, a classifier trained on a selected subset of six QUS features derived from the original RF data achieved a test accuracy of 82 ± 6%. In training and testing the classifier with the same subset of QUS features derived from the synthetic RF data, the deep ViT cGAN matched the original model’s performance (accuracy = 82 ± 6%), outperforming the Pix2Pix and shallow ViT cGANs. When the feature selection and classifier training and testing were exclusively performed on the synthetic QUS parameters, the Deep ViT cGAN (accuracy = 81 ± 7%) and Pix2Pix cGAN (accuracy = 81 ± 6%) demonstrated competitive performance, while the Shallow ViT remained slightly lower (accuracy = 79 ± 6%). The promising results obtained in this study demonstrate the feasibility of RF data synthesis from B‐mode images, and therefore, is a step forward towards QUS‐based tissue characterization without the necessity of direct access to RF data.

## Introduction

According to the World Health Organization, breast cancer is the second most commonly diagnosed cancer worldwide, with about 2.5 million new cases reported in 2022^1^. In women, breast cancer is the most prevalent type of cancer, both in terms of the number of reported cases and cancer-related deaths^1^. Early detection of breast cancer is essential for effective treatment planning and improving therapy outcomes and patient prognosis. The screening protocol for breast cancer diagnosis typically initiates with x-ray mammography for detecting abnormalities, followed by standard ultrasound (B-mode imaging) and/or dynamic contrast-enhanced magnetic resonance imaging (DCE-MRI) for further evaluation of the abnormalities. In case of suspicious lesions on ultrasound or MRI, biopsy is performed as the gold standard for confirming malignancy and determining tumor grade. However, biopsy is an invasive and painful procedure that also poses a risk of tumor cell migration^2^. Moreover, due to low specificity of ultrasound B-mode images patients may undergo unnecessary biopsies^3^. DCE-MRI has better specificity in characterizing breast lesions but it is relatively expensive, and not always available for rapid diagnosis due to longer waiting times and limited accessibility^4^. These challenges highlight the importance of developing imaging techniques that are inexpensive and accessible for rapid diagnosis, while accurate in characterizing lesions.

In recent years, quantitative ultrasound (QUS) has gained increasing attention as a promising tool for cancer detection and lesion characterization due to its advantages such as being non-invasive, cost-effective, and accessible^5^. Unlike conventional B-mode ultrasound imaging, which primarily relies on the envelope of echo signals to generate grayscale images, QUS analyzes raw radiofrequency (RF) data to extract tissue-specific acoustic properties^6^. By utilizing spectral parameters such as the mid-band fit (MBF), spectral slope (SS), and spectral intercept (SI), QUS enables a more detailed assessment of tissue microstructure^7^. QUS techniques have demonstrated their capabilities across various applications, including assessing bone structures to diagnose osteoporosis^8,9^, characterizing tissue types in the liver, prostate, and thyroid^10–13^, evaluating tumor response to treatment in preclinical and clinical models^14–17^, distinguishing blood clot constituents^18^, and detecting metastatic lymph nodes in vivo^19^.

In the context of breast cancer, early preclinical studies using animal models have highlighted the potential of QUS backscatter parameters, specifically effective scatterer diameter (ESD) and effective acoustic concentration (EAC), in distinguishing benign mammary fibroadenomas from malignant 4T1 carcinomas^20^ as well as differentiating between breast carcinomas and sarcomas^21^. These findings were reinforced later by studies which used QUS parametric maps and their texture features to better distinguish benign and malignant breast lesions^22–25^. In another line of studies, researchers have explored the use of QUS techniques to assess breast cancer response to neoadjuvant chemotherapy (NAC) within few weeks after treatment initiation^16,17,26,27^. These studies have shown strong correlations between early changes in QUS parametric images and the clinical response observed at the end of therapy. Recent studies have demonstrated the potential of QUS-based radiomics to characterize intra-tumoral heterogeneity in locally advanced breast cancer and to predict response to neoadjuvant chemotherapy prior to treatment initiation^28,29^.

One limitation associated with QUS imaging is that it requires access to raw RF data from the scanned tissue, which is not readily available on most clinical ultrasound systems. Unlike B-mode images, which are processed and compressed for efficient transmission and storage, RF data consists of high-resolution, uncompressed signals sampled at high frequencies, requiring more processing time and memory, transmission bandwidth, and storage space. Therefore, even the RF-enabled scanners discard the acquired RF data at early stages of B-mode image generation process during clinical scans, unless the system operates at specific research modes. This limitation has hindered the real-time QUS imaging capabilities and remains a barrier for widespread clinical adoption of QUS techniques.

Over the past decade, generative artificial intelligence (AI) has emerged as a powerful tool in medical image synthesis. Techniques based on generative adversarial networks (GANs)^30^, variational autoencoders (VAEs)^31^, and other CNN-based models like U-net^32^ have been successfully applied to a variety of image synthesis tasks, including both cross- and within-modality translation, e.g., MRI to CT translation or translation between different MRI sequences^33,34^, super-resolution imaging, e.g., synthesizing 7T MRI images from 3T^35^, artifact correction^36^ and data augmentation^37,38^. Conditional GANs (cGANs) such as Pix2Pix^39^, and cycle-consistent GANs^40^ have particularly shown effective in paired and unpaired image translation, respectively. More recently, vision transformer-based generative models^41,42^ and denoising diffusion probabilistic models (DDPMs)^43,44^ have also been used increasingly in medical image synthesis applications. In the domain of breast cancer imaging, generative AI has been applied to simulate mammograms^45^, reconstruct ultrasound images^46^, and generate synthetic augmentation data to improve lesion detection and classification models^47^, addressing data availability and acquisition constraints.

This study aimed at addressing a key limitation in QUS imaging, i.e. RF data inaccessibility, by synthesizing RF signals from the associated B-mode images using conditional generative adversarial networks (cGANs). Three generative models including a Pix2Pix cGAN, a shallow Vision Transformer (ViT) cGAN and a deep ViT cGAN were developed and evaluated on a large dataset of paired RF/ B-mode frames acquired from breast lesions. The fidelity of the synthetic RF data was assessed via several sample-level metrics and a downstream benign/malignant classification task using QUS parametric imaging. The results demonstrated the feasibility of synthesizing RF data with high similarity to their original counterparts, and the efficacy of the corresponding QUS parametric images in breast lesion characterization.

## Materials and methods

### Data acquisition and preprocessing

This study was conducted in accordance with institutional research ethics board approval at Sunnybrook Health Sciences Centre and York University, Toronto, ON, Canada. All methods were performed in accordance with the relevant guidelines and regulations. Ultrasound RF data and B-mode images were collected from 152 patients with suspicious breast lesions at the Rapid Diagnostic Unit (RDU) of Louise Temerty Breast Cancer Centre at Sunnybrook Health Sciences Centre, following informed consent. Data acquisition was performed using a Sonix Touch system (Ultrasonix, Vancouver, Canada) utilizing a linear array transducer (L14-5/60 W) operating at a nominal frequency of 10 MHz. RF data were digitized with a 40 MHz sampling rate and collected along 510 lateral scan lines, corresponding to a 6 cm lateral field of view and a 4 cm imaging depth. Multiple RF/B-mode pairs were acquired at 4 different imaging planes spanning the lesion volume as well as through a single continuous sweep over the entire lesion.

Among 152 patients, 77 and 75 patients were confirmed with benign and malignant lesions, respectively, based on pathology and radiology reports. For developing the generative models, the dataset was split at the patient level into training (98 patients; 50 benign and 48 malignant lesions), validation (16 patients; 8 benign and 8 malignant lesions), and independent test (38 patients; 19 benign and 19 malignant lesions) sets using a stratified random splitting. In terms of the number of frames included, the training set contained 21,174 RF/B-mode pairs, the validation set included 3,456 pairs, and the test set comprised 8,919 pairs. The RF frames contained 2080 samples × 510 lines, while the B-mode images were of size 373 × 541 pixels. For preprocessing, the B-mode images were resized to 2080 × 510 pixels using linear interpolation and flipped along the y-axis to correct for the inversion applied by the scanner. To make the size of both the B-mode images and RF frames a power of two, simplifying the patch partitioning and up-sampling and down-sampling through the models, the first and last column of each array were repeated in place, bringing the number of columns to 512. Additionally, the last 32 rows (92 μm) were omitted to obtain 2048 rows for both RF frames and B-mode images. The RF frames and B-mode images were subsequently normalized to the range [0,1] using a min-max normalization with global parameters obtained from the training set.

For the QUS analysis and lesion classification task, the test set was kept the same for independent evaluation, while the training and validation sets were combined to perform 5-fold cross-validation for feature selection and classifier optimization. The QUS parametric maps were generated for each lesion from the RF data associated with multiple imaging planes spanning the lesion volume. The regions of interest (ROIs) for each lesion were manually delineated on the corresponding ultrasound images under the guidance of an experienced radiologist and cross-validated using the lesion size and characteristics reported from MRI findings. The QUS mean values and texture features (described in section D) were computed for all ROIs associated with each lesion and averaged across the lesion volume before analysis by the classifier.

### Models

**Fig. 1:**
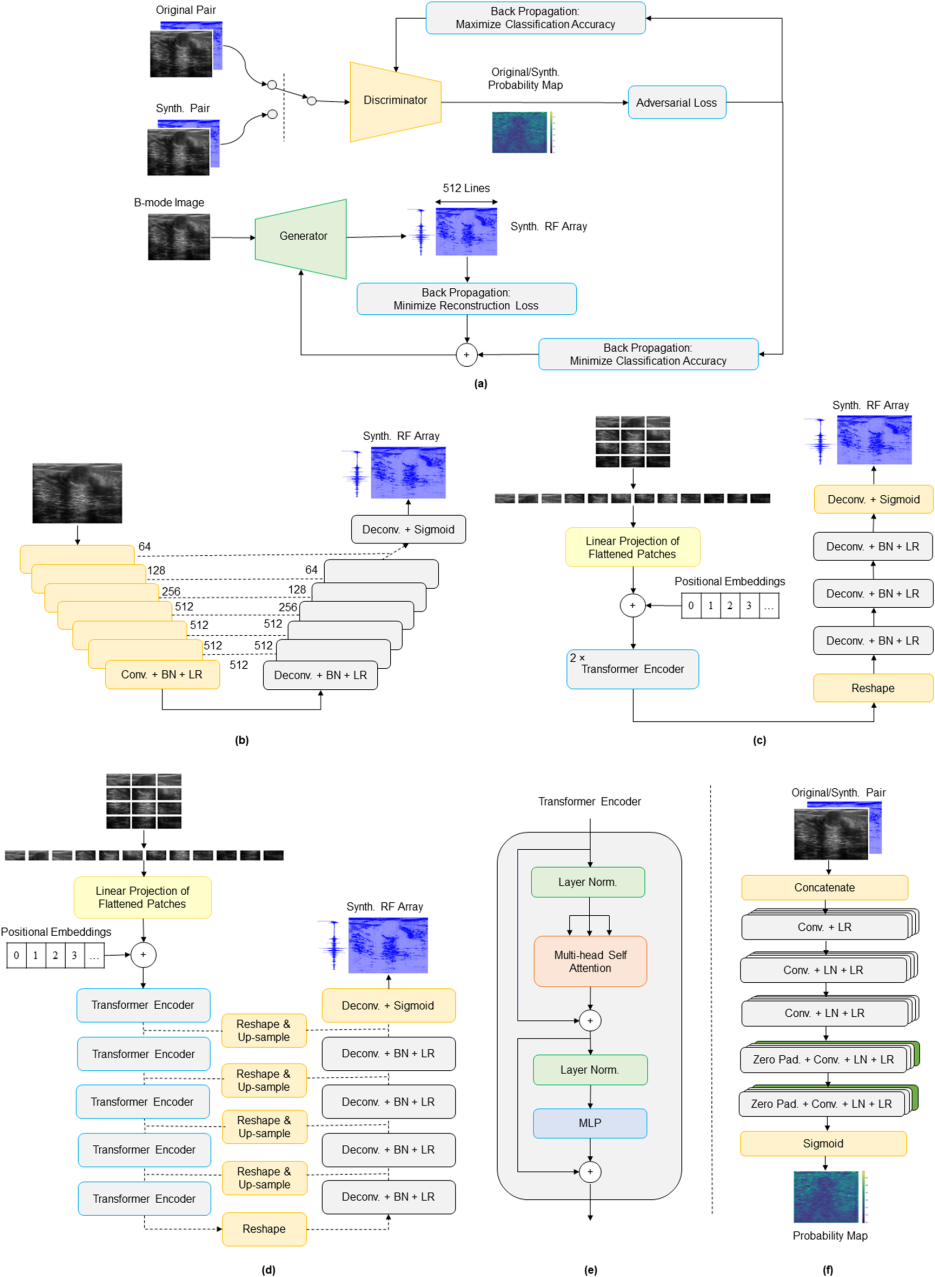
(**a**) Overall architecture of the conditional GAN framework for synthesizing RF ultrasound data from B-mode images. Generator architectures for (**b**) Pix2Pix cGAN, (**c**) Shallow ViT cGAN, and (**d**) Deep ViT cGAN. (**e**) Transformer encoder configuration used in ViT-based generators. (**f**) Discriminator architecture. Conv: Convolution, Deconv: Deconvolution, BN: Batch Normalization, LR: Leaky Relu, LN: Layer Normalization.

Three different conditional GANs were developed and evaluated for translating B-mode images back into their corresponding RF data. Unlike traditional GANs^30^, which learn mapping of an input random noise vector *z* to the output domain *y* during the training process, cGANs^39^ learn how to transform an input domain *x* (in this case, B-mode image) to a target domain *y* (RF data). Each cGAN model comprises a generator *G*, that is trained to synthesize RF data conditioned on the input B-mode image, and a discriminator *D*, which is trained adversarially to distinguish between original and generated RF samples (Fig. 1).

The training objective is to optimize both networks such that *G* synthesizes outputs that are not only visually realistic but also indistinguishable by *D* from original RF data. In this study, a patch-based discriminator was employed for all the three cGAN models, while the generator architecture varied across them (Fig. 1). The selection of generator architectures and their configurations was guided by prior literature and targeted preliminary experiments. Pix2Pix was chosen as a widely adopted baseline for paired image-to-image translation tasks in medical imaging. Building on this network, alternative generator designs, including conventional encoder-decoder networks, ResNet-based models, and ViT-based architectures, were explored to assess whether increased model capacity could improve RF synthesis. Based on these preliminary evaluations, three representative architectures were selected for systematic comparison: a U-Net-based Pix2Pix generator, a shallow ViT-based generator, and a deeper ViT-based generator. Diffusion-based generative models were also explored; however, due to their stochastic nature and reduced ability to preserve strict input-output correspondence, they were excluded from the present study. Further details about the generator and discriminator architectures have been provided in the following sections.

#### Pix2Pix Generator

In this model, a U-Net-based encoder-decoder architecture^32^ was used to map ultrasound B-mode images to RF data. The model takes a single-channel input of size 2048 × 512 pixels and outputs an array of the same dimensions. The encoder consists of seven down-sampling convolutional layers with increasing filter sizes (64 to 512), using 4 × 4 kernels and a stride of 2. Following the convolutional layers, batch normalization and Leaky ReLU activation are applied. The decoder mirrors the encoder with six up-sampling transposed convolutional layers, applying dropout in the early stages and using ReLU activation. Skip connections link the corresponding encoder and decoder layers to facilitate gradient flow. A final up-sampling transposed convolution layer with sigmoid activation produces the output in the range [0,1]. The encoder/decoder depth was evaluated using both 7 and 9-level down/up-sampling configurations, in line with the deep architecture of the original Pix2Pix work. As the quantitative performance differences were marginal, the 7-level configuration was selected due to lower architectural complexity and improved preservation of spatial texture in reconstructed B-mode images.

#### Shallow ViT-based Generator

This generator model was designed using a vision transformer-based architecture to reconstruct RF data from encoded B-mode patches. The model begins by splitting the input B-mode image into non-overlapping patches of size 16 × 16 pixels, a patch size in line with standard ViT architectures, resulting in 4096 patches per RF frame. These patches were then flattened, linearly projected, and combined with positional embeddings. These patch tokens pass through two transformer encoder blocks, each consisting of multi-head self-attention followed by a feed-forward MLP with residual connections and layer normalization, to capture essential contextual relationships. The resulting feature embedding is reshaped into a low-resolution feature map, which is then up-sampled via a decoder of four transposed convolution layers. The first three deconvolutions include batch normalization and Leaky ReLU activations, while the final transposed convolution uses a sigmoid activation to constrain the synthesized RF signal samples between 0 and 1.

#### Deep ViT-based Generator

The deep ViT GAN employs the same initial patch-embedding and positional-encoding strategy, i.e., dividing the input B-mode image into non-overlapping 16 × 16- pixel patches, linearly projecting them, and adding positional embeddings, but processes the tokens through five stacked transformer encoder blocks to capture richer long-range dependencies and contextual information. The encoded representation is then reshaped and progressively up-sampled using transposed convolution layers. To enhance spatial resolution and retain fine-grained details, skip connections with reshaping and up-sampling blocks are used to connect earlier transformer layers to the decoder layers. The final RF reconstruction is obtained through a sigmoid-activated transposed convolution layer, ensuring the output is scaled between 0 and 1. The use of five transformer encoder blocks in the deep ViT configuration was motivated by the need to increase model capacity for learning more robust features while remaining within available GPU memory constraints.

#### Discriminator

The discriminator was designed to classify the authenticity of local regions (patches) in the RF data, rather than labeling the entire array as original or synthetic. It receives two single-channel inputs, including a B-mode image and its corresponding RF data (either original or synthetic), both with dimensions of 2048 × 512. These inputs are concatenated along the channel dimension and passed through three down-sampling blocks. Each down-sampling block consists of a 3 × 3 convolutional layer with stride 2 and increasing filter sizes (64, 128, 256), followed by layer normalization and Leaky ReLU activation. The output is then processed by two 3 × 3 convolutional layers with stride 1 and filter sizes of 512 and 1, respectively, each preceded by zero-padding. Layer normalization and Leaky ReLU are applied after the first convolution, and a sigmoid activation followed the final convolution to produce a single-channel output probability map of size 256 × 64. This map represents the probability of each patch being original or synthetic. In a set of preliminary experiments, deeper discriminator configurations were also explored. However, increasing discriminator depth led to training instability due to the discriminator overpowering the generator. Therefore, the original patch discriminator depth suggested by the Pix2Pix cGAN was retained and used consistently across all generator variants to ensure a fair comparison.

### Training configurations

To train both the generator and discriminator models, an adversarial loss function based on binary cross-entropy (BCE) was employed to ensure realistic RF reconstructions. Label smoothing was applied to the discriminator target labels to mitigate overconfidence and improve training stability. The generator was also guided by L1 and L2 reconstruction losses, weighted at 1000 and 100, respectively, to encourage generating outputs that are not only realistic but also closely aligned with the ground-truth RF data for each sample. An Adam optimizer was used for all the three cGAN models with a batch size of 1, and a learning rate of 1e-4 for the generator and 1e-5 for the discriminator. A weight decay of 1e-4 was applied, while the β1 and β2 parameters were set to 0.5 and 0.999, respectively. The initial values for these hyperparameters were chosen based on commonly used settings for GAN training and subsequently fine-tuned to ensure stable training and preventing the generator/discriminator model from overpowering the other one. The models were first trained for 150 epochs where the resulting B-mode images from the generated RF data visually resembled the original B-mode images. The training then continued for 10 additional epochs, during which the validation set was used to select the model with the lowest reconstruction loss.

### QUS parametric imaging and analysis

Parametric maps of MBF, SS, SI, ESD, and EAC were generated within the ROI (tumor core) and a 5-mm margin around it in each imaging plane^24,25^ using the original and synthetic RF data separately. A sliding window analysis was applied using hanning-gated windows of size 2 × 2 mm, with an overlap of 94% in both axial and lateral directions. The mean power spectrum of the RF lines within each window was calculated using the Fast Fourier Transform (FFT), and normalized using the mean power spectrum of a reference phantom within the same window to compensate for system transfer functions and transducer beam-forming effects. The reference data for normalization was obtained under identical scan settings from a tissue-mimicking phantom. The phantom was composed of 5–30 μm glass beads suspended in a homogeneous medium of microscopic oil droplets embedded in gelatin (University of Wisconsin, Department of Medical Physics, Madison, WI, USA). Its measured acoustic properties included an attenuation coefficient of 0.786 dB/MHz/cm and a speed of sound of 1540 m/s. A two-layer attenuation correction, intervening tissue and tumor, was performed using the point-compensation method^48^ based on the total attenuation estimation. The attenuation coefficient (ACE) of the tumor was estimated using a spectral difference method^49^ by measuring the rate of change in spectral power magnitude with depth and frequency relative to the reference phantom. An attenuation coefficient of 1 dB/MHz.cm was applied for the intervening breast tissue based on ultrasound tomography measurements^50,51^. Parametric maps of MBF, SS and SI were generated using a linear regression analysis on the attenuation-corrected normalized power spectrum within each analysis window^52^. The ESD and EAC parameters were calculated within each window by fitting a spherical Gaussian form-factor model to the backscatter coefficient (BSC)^53^, which was estimated using the attenuation-corrected normalized power spectrum.

In addition to the mean-value parameter derived from the parametric maps, texture analysis was conducted using the gray-level co-occurrence matrix (GLCM)^54^ to quantify heterogeneities within the tumor core and the surrounding tissue^24^. The GLCM captures second-order statistical information by examining the spatial relationship between neighboring pixels in an image. The full range of gray-level intensities in each parametric image was linearly scaled and quantized into 16 levels. Symmetric GLCMs were computed at inter-pixel distances of 1, 2, 3, 4, and 5 pixels, and at four angular directions (0°, 45°, 90°, and 135°). Four texture features including contrast, correlation, energy, and homogeneity^24^ were extracted from each GLCM and averaged over all GLCMs of each parametric image to quantify image texture.

### Evaluation

To assess the quality of the synthetic RF data, two approaches were pursued, including sample-level evaluation as well as comparing the corresponding QUS parametric maps and their performance in breast lesion classification. The sample-level evaluation metrics included the normalized root mean squared error (NRMSE), normalized mean absolute error (NMAE), structural similarity index (SSIM), and peak signal to noise ratio (PSNR)^55^. The NRMSE, NMAE, and SSIM were calculated for the synthetic RF data after denormalization. Additionally, the SSIM and PSNR were estimated for the B-mode images reconstructed from the synthetic RF data.

Beyond sample-level evaluations, qualitative and distributional analyses were conducted to evaluate the similarity of the QUS features derived from synthetic and original RF data. Specifically, uniform manifold approximation and projection (UMAP) method^56^ was applied to project the high-dimensional QUS feature arrays into a two-dimensional space and to visualize the overall distribution patterns of synthetic versus original features. In addition, histograms with kernel density estimates were generated for individual QUS parameters to compare the central tendency and spread of feature distributions.

The performance of the QUS mean-value and texture features derived from the synthetic and original parametric maps were subsequently evaluated in classifying benign versus malignant breast lesions and compared. The QUS features were processed through a two-step feature selection procedure on the training set to remove redundant and irrelevant features that do not contribute to classification. First, the mutual information criterion^55^ was applied to identify the 20 most informative features for lesion classification. These features were then passed to a logistic regression model with an elastic net penalty, used as a wrapper method for further reducing the number of the features^57^. The elastic net combined L1 (Lasso) and L2 (Ridge) regularization with weights of 0.7 and 0.3, respectively. The stochastic average gradient accelerated (SAGA) solver was used with a maximum of 10,000 iterations to ensure convergence. In this setup, L1 regularization shrinks the coefficients of irrelevant features to zero (feature selection effect), while L2 regularization stabilizes the model by shrinking coefficients without eliminating them (model stabilization effect). A threshold equal to the mean of the absolute coefficients was applied, retaining only features with coefficients above this value. After feature selection, the classification modeling was performed using a support vector classifier (SVC)^55^ with an RBF kernel. The optimal SVC parameters (C and γ) were determined through grid search, using stratified 5-fold cross-validation on the training set with the area under the receiver operating characteristic (ROC) curve (AUC) as the performance metric. The optimized model, which was trained on the entire training set, was then evaluated on the unseen test set using bootstrapping technique with 1,000 resamples to assess performance variability on the test set.

Four separate model experiments were conducted for comparison. In the first experiment, the feature selection, classifier training and evaluation were performed using the QUS parametric maps generated from the original RF data. In the second experiment, the entire process (feature selection, classifier training and evaluation) was performed using the QUS parametric maps generated from the synthetic RF data. In the third, the classifier training and evaluation were performed using the QUS maps generated from the synthetic RF data, but the same QUS features selected in experiment 1 were applied. Finally, in the fourth experiment, feature selection and classifier training were done using the original-RF-derived QUS maps while the evaluation was performed on the synthetic-RF-derived features.

## Results

Table 1 summarizes sample-level quantitative comparisons between the original and synthetic RF data on the independent test set. The three cGAN models demonstrated similar results in terms of sample-level metrics, while the Deep ViT cGAN achieved slightly higher SSIM scores on the B-mode images generated from its synthetic RF data. Compared to the shallow ViT cGAN, the Pix2Pix cGAN and deep ViT cGAN models delivered a slightly lower NRMSE on the synthetic RF data as well as a slightly higher PSNR on the corresponding B-mode images. However, the shallow ViT cGAN demonstrated on par NMAE and SSIM scores on the synthetic RF data, implying its competitive performance compared to the other models.

**Table 1:** Quantitative evaluation of synthetic RF data against their original counterparts at sample level for the three cGAN architectures. Values are reported as mean ± standard deviation across the test set.

Model	NRMSE (RF)	NMAE (RF)	SSIM (RF)	SSIM (B-mode)	PSNR (B-mode)
Pix2Pix cGAN	2.8 ± 0.7%	1.4 ± 0.3%	0.82 ± 0.05	0.88 ± 0.06	33.4 ± 1.7 dB
Shallow ViT cGAN	2.9 ± 0.7%	1.4 ± 0.3%	0.82 ± 0.05	0.87 ± 0.04	32.8 ± 1.8 dB
Deep ViT cGAN	2.8 ± 0.6%	1.4 ± 0.3%	0.82 ± 0.05	0.89 ± 0.02	33.3 ± 1.7 dB

**Fig. 2:**
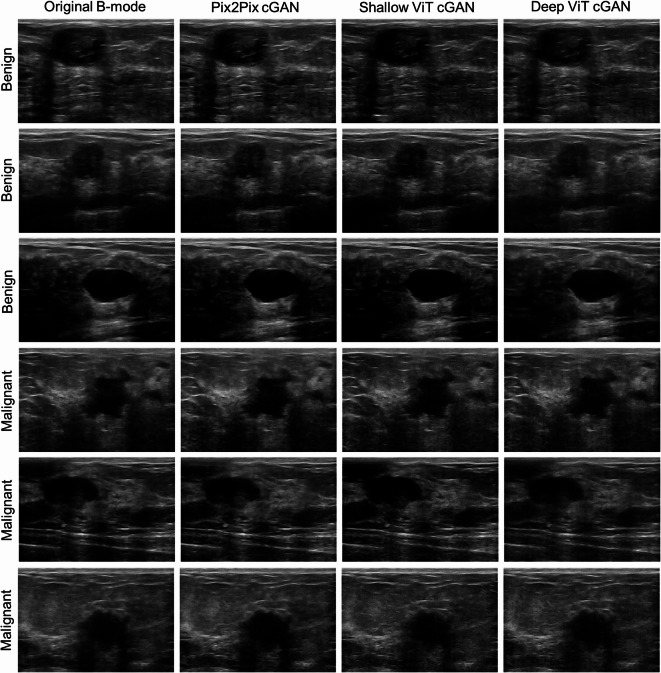
B-mode images generated from the original and synthetic RF data for representative benign and malignant lesions.

Figure 2 presents the B-mode images generated from the original and synthetic RF data for representative benign and malignant lesions. Although subtle differences in speckle texture and edge sharpness are visible in the images generated from the synthetic data, overall, all models faithfully preserved the lesion morphology and speckle pattern of the original data. The original and synthetic RF data were subsequently used to generate QUS parametric maps for characterizing benign versus malignant lesions.

**Fig. 3:**
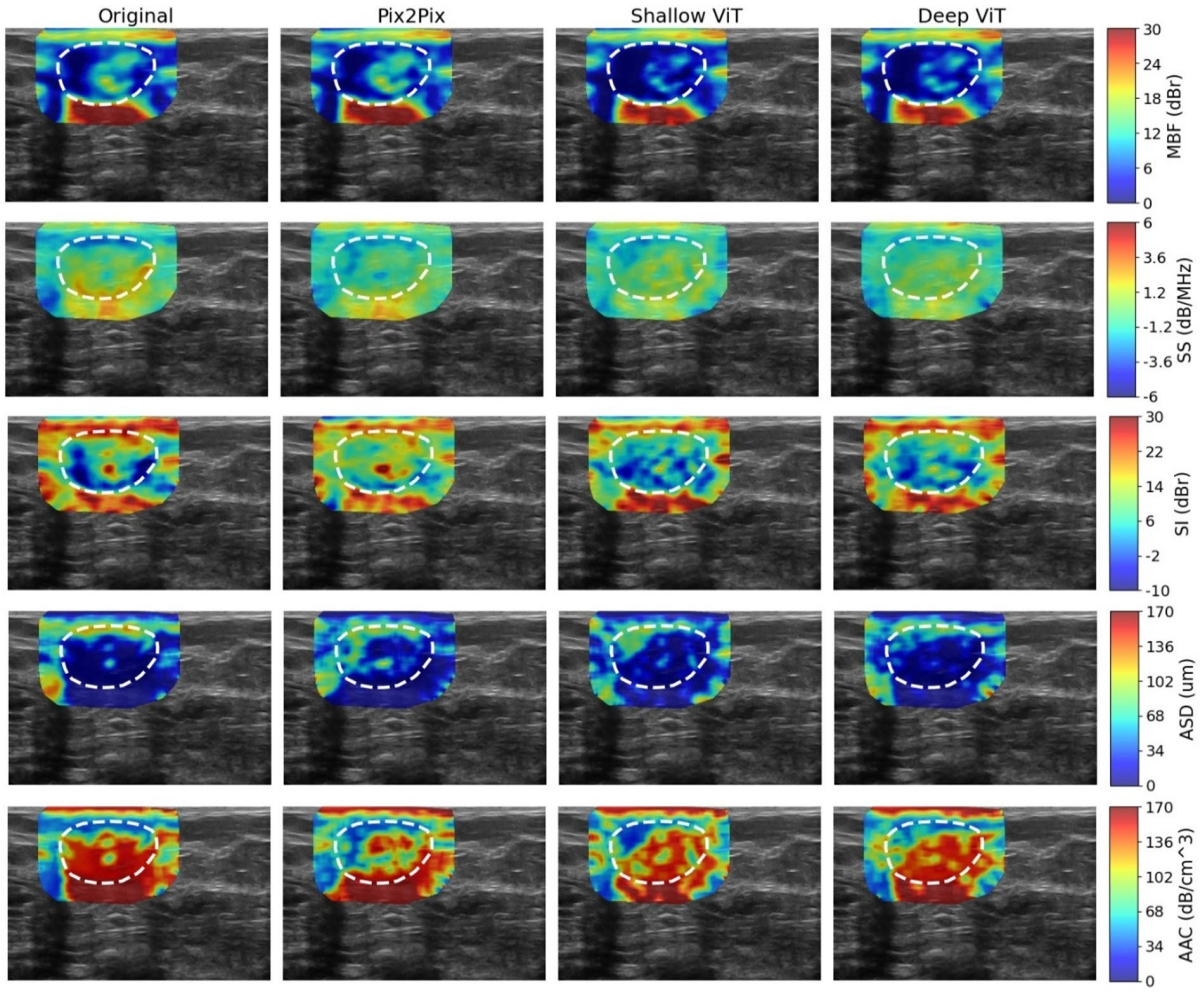
QUS parametric maps generated from the original and synthetic RF data for a representative benign lesion. The maps are overlaid on the corresponding B-mode images.

**Fig. 4:**
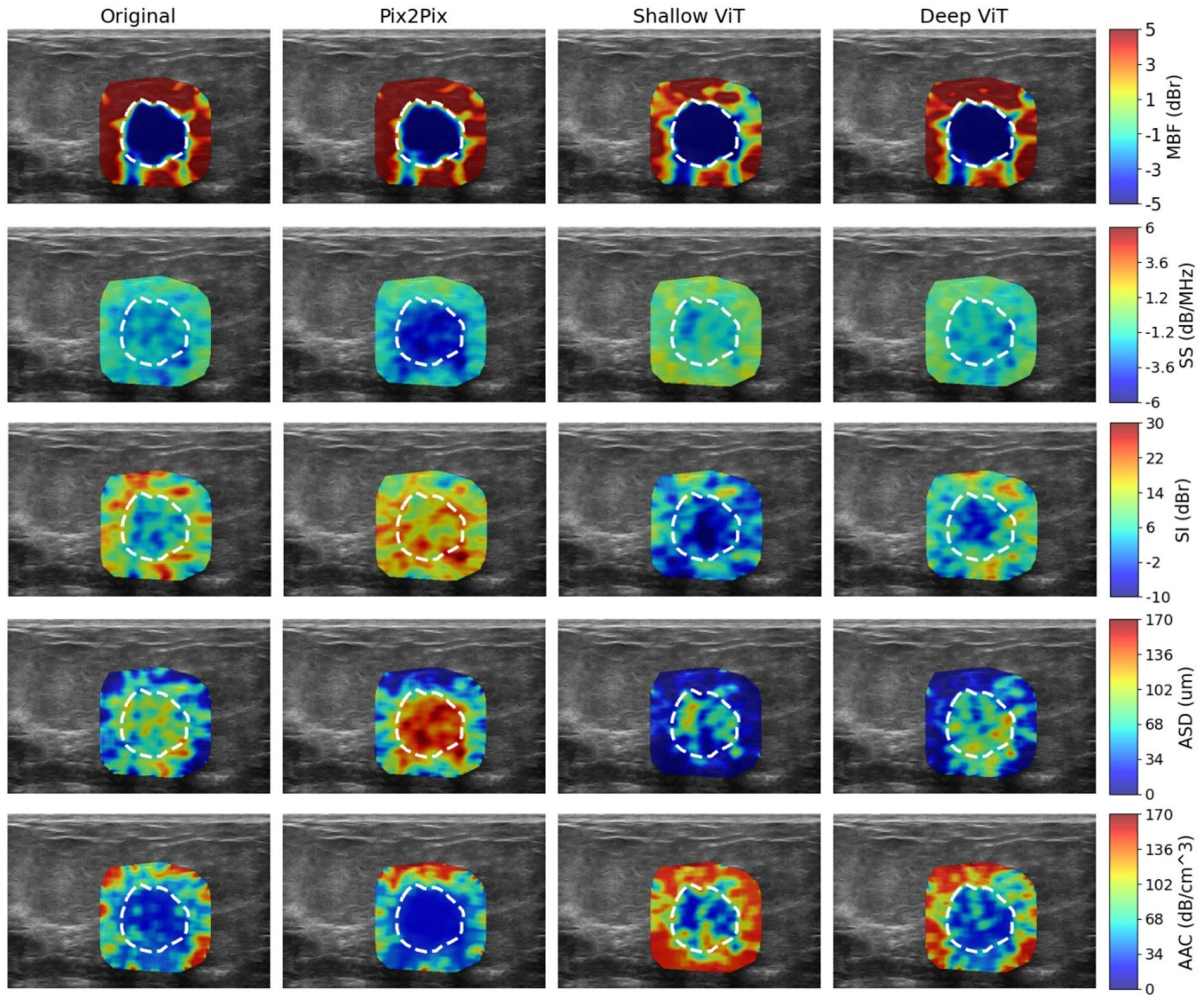
QUS parametric maps generated from the original and synthetic RF data for a representative malignant lesion. The maps are overlaid on the corresponding B-mode images.

Figures 3 and 4 illustrate the QUS parametric maps derived from the original and synthetic RF data, for representative benign and malignant lesions from the test set. Among the parametric maps associated with the Pix2Pix and shallow ViT cGAN models, the MBF and SS maps more closely matched the original ones. The deep ViT cGAN, however, demonstrated superior overall fidelity, generating synthetic RF data that yielded QUS maps that most closely resemble those derived from the original RF data.

Table 2 reports the NMAE for the synthesized RF data and the corresponding QUS parametric maps, computed at pixel level within the lesion core and its 5 mm margin across the test set and normalized by the global dynamic range of each parameter. The RF synthesis errors were low across all models, indicating decent recovery of the underlying RF signal within the ROI region. Importantly, these RF-level discrepancies translated into relatively larger errors in downstream QUS parameters, with the magnitude of error varying by parameter type. The parameters that depend on first-order spectral characteristics (MBF, SS, SI) exhibited relatively lower errors compared to the parameters derived from higher-order and nonlinear spectral characteristics (ASD, AAC), reflecting increased sensitivity to RF perturbations in the latter parameters. Among the evaluated models, the Deep ViT cGAN achieved the lowest errors across all parameters, implying a more accurate synthesis of RF signals.

**Table 2:** Normalized mean absolute error (NMAE) of the synthesized RF data and derived QUS parametric maps within the lesion core and its 5-mm margin. Values are reported as mean ± standard deviation across the test set and normalized by each parameter’s global dynamic range.

Model	RF	MBF	SS	SI	ASD	AAC
Pix2Pix cGAN	1.3 ± 0.6%	2.6 ± 1.7%	5.0 ± 2.0%	6.3 ± 2.2%	8.5 ± 2.7%	7.5 ± 3.1%
Shallow_ViT cGAN	1.3 ± 0.6%	2.8 ± 1.5%	5.2 ± 2.1%	6.4 ± 2.2%	8.6 ± 2.6%	7.6 ± 3.0%
Deep_ViT cGAN	1.2 ± 0.6%	2.4 ± 1.1%	4.8 ± 2.0%	6.1 ± 2.1%	8.2 ± 2.6%	7.2 ± 3.1%

**Fig. 5:**
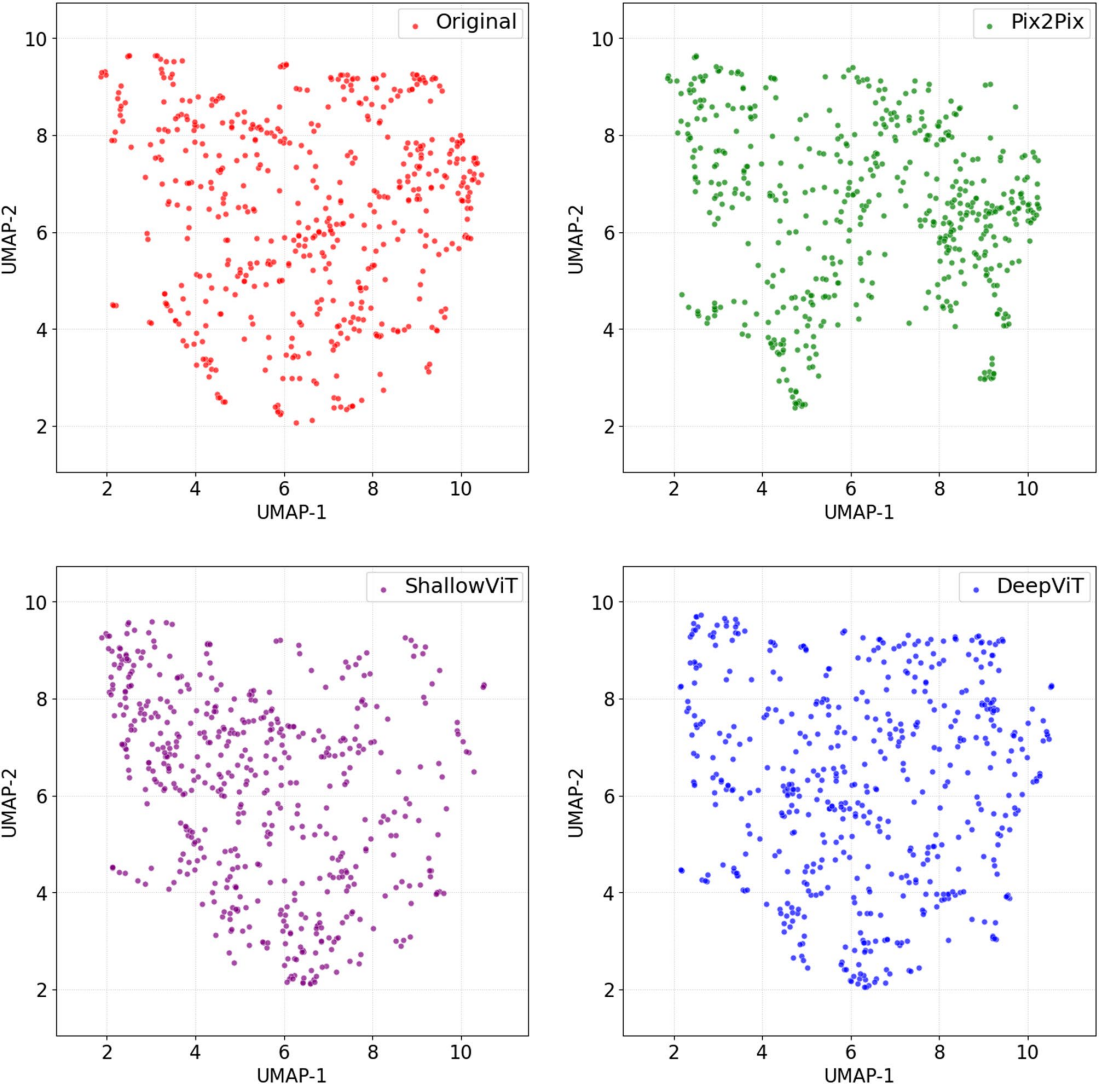
UMAP projections of QUS feature arrays derived from the original RF data (top left) and synthetic RF data generated using Pix2Pix (top right), Shallow ViT (bottom left), and Deep ViT (bottom right) cGAN models.

To qualitatively evaluate the similarity between QUS feature distributions derived from the original and synthetic RF data, the UMAP method was applied to project the high-dimensional QUS feature arrays into a two-dimensional space. The resulting embeddings are shown in Fig. 5. Each point in the UMAP projections here embeds a 25-dimensional QUS feature vector derived from five parametric maps, including the mean-value and four texture features per map. When synthetic features closely match the original features in the high-dimensional space, their low-dimensional UMAP projections are expected to exhibit similar spatial organization and overlap. As shown in Fig. 5, the overall distribution patterns of the synthetic feature point clouds closely resemble those of the original features, particularly for the Deep ViT cGAN. This alignment suggests that the generative models were successful in producing RF data with associated QUS features reflecting the underlying distribution of the original feature set.

Although UMAP is primarily a visualization technique and not intended as a quantitative evaluation tool, relative distance-based measures in the embedded space can potentially provide insight into the alignment between the synthetic and original QUS feature distributions. Accordingly, we quantified the average distance between synthetic and original feature embeddings in the UMAP space. The Deep ViT cGAN exhibited the lowest embedding discrepancy (UMAP-MAE = 1.0 ± 0.8), followed by the Pix2Pix cGAN (UMAP-MAE = 1.2 ± 0.9) and Shallow ViT cGAN (UMAP-MAE = 1.3 ± 0.9). These distance-based trends are consistent with the qualitative observations in Fig. 5, where the Deep ViT model demonstrates the strongest overlap with the original feature distribution.

**Fig. 6:**
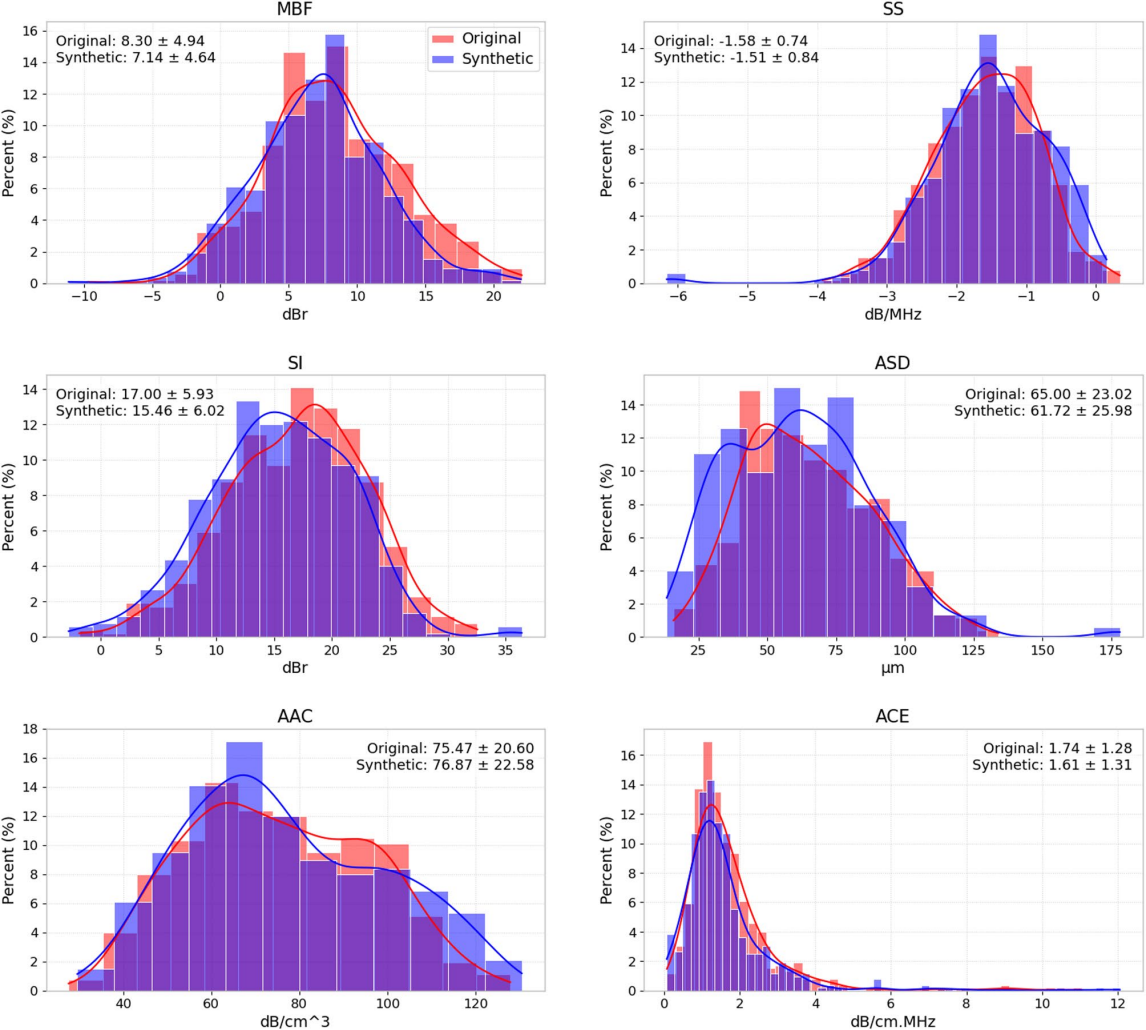
Histograms with overlaid kernel density estimates of mean-value QUS features, comparing original features with synthetic features derived from Deep ViT-generated RF data. Annotation boxes report the mean ± standard deviation for each feature.

Complementing the UMAP visualization, Fig. 6 presents histograms of individual QUS mean-value features from the original data and those derived from Deep ViT synthesized RF data. The histogram distributions show a good agreement between the original and synthetic features, particularly in their central tendency and overall spread.

In the next step, the efficacy of the synthetic RF data was evaluated via a downstream benign versus malignant classification task using the associated QUS features. Table 3 summarizes the results of the four separate experiments conducted. In the first experiment, where the feature selection and the classifier training and evaluation were performed using the QUS parametric maps associated with the original RF data, the classifying model achieved an accuracy of 82 ± 6%, sensitivity of 84 ± 8%, and specificity of 79 ± 9% on the unseen test set. In the next experiment, feature selection as well as classifier training and testing were entirely conducted using the synthetic QUS data. The RF data synthesized by Pix2Pix cGAN and Deep ViT cGAN both resulted in an accuracy of 81 ± 6–7%, with a sensitivity of 84 ± 8% and a specificity of 79 ± 9%, closely matching the original data performance. The synthesized RF data from Shallow ViT cGAN led to slightly lower accuracy (79 ± 6%) with a sensitivity of 84 ± 8% and a specificity of 74 ± 10%.

**Table 3:** Results of benign versus malignant classification on the independent test set using the original QUS features as well as the synthetic QUS features through three experiments with different dependency conditions relative to the original QUS features.

Model	Test Acc.	Test Sens.	Test Spec.	Test Prec.	Selected Features
Training on original QUS → testing on original QUS					
Original QUS	82 ± 6%	84 ± 8%	79 ± 9%	81 ± 7%	MBF-COR, AAC-CON, SI-CON, SI-HOM, MBF-CON, SS-HOM
Training & testing on synthetic QUS					
Pix2Pix cGAN	81 ± 6%	84 ± 8%	79 ± 9%	80 ± 7%	**MBF-COR**,** SI-CON**, **MBF-CON**, SI-COR, SS-COR, SS-ENE, MBF-HOM,
Shallow ViT cGAN	79 ± 6%	84 ± 8%	74 ± 10%	77 ± 7%	**MBF-CON**,** MBF-COR**, ASD-CON
Deep ViT cGAN	81 ± 7%	84 ± 8%	79 ± 9%	80 ± 7%	**AAC-CON**,** MBF-COR**, **MBF-CON**, ASD-HOM, AAC-ENE, SI
Training & testing on synthetic QUS (best features based on original QUS)					
Pix2Pix cGAN	79 ± 6%	84 ± 8%	74 ± 10%	77 ± 7%	---
Shallow ViT cGAN	81 ± 6%	79 ± 9%	84 ± 8%	84 ± 8%	---
Deep ViT cGAN	82 ± 6%	84 ± 8%	79 ± 9%	81 ± 7%	---
Training on original QUS → testing on synthetic QUS					
Pix2Pix cGAN	76 ± 7%	79 ± 9%	74 ± 10%	75 ± 7%	---
Shallow ViT cGAN	79 ± 7%	79 ± 9%	78 ± 9%	79 ± 8%	---
Deep ViT cGAN	84 ± 6%	84 ± 8%	84 ± 9%	84 ± 7%	---

Note that “original QUS features” and “synthetic QUS features” refer to features derived from original and synthetic RF data, respectively. The selected synthetic features matching those selected from the original QUS feature set are indicated in bold. Acc.: accuracy, Sens.: sensitivity, Spec.: specificity, Prec.: precision, SS: spectral slope, MBF: mid-band fit, ASD: average scatterer diameter, SI: spectral intercept, COR: correlation, CON: contrast, HOM: Homogeneity, ENE: energy.

In the third experiment, using the best-performing feature subset selected based on the original QUS maps in the first experiment, the classifier was trained and tested on the synthetic QUS data. Here, the synthetic RF data from Deep ViT cGAN matched the performance of the original RF data (first experiment) with an accuracy of 82 ± 6%, a sensitivity of 84 ± 8%, and a specificity of 79 ± 9%. The Shallow ViT GAN and Pix2Pix GAN resulted in a slightly lower performance with accuracies of 81 ± 6% and 79 ± 6%, respectively. In the final set of experiments where the classifier was trained on the QUS features extracted from the original RF data and tested on those derived from the synthetic RF data, the performances declined except for the data generated by Deep ViT cGAN (accuracy = 84 ± 6%). Although the Deep ViT cGAN achieved a slightly higher mean accuracy, given the reported standard deviations, it still lies within the variability range of the original QUS performance.

## Discussion and conclusion

This study investigated for the first time the feasibility of synthesizing ultrasound RF data from B-mode images for QUS analysis and breast tissue characterization applications. QUS techniques extract tissue-specific biomarkers directly from the raw RF signals to reveal microstructural properties that may not be consistently detectable from conventional B-mode images. Common QUS approaches include spectral and backscatter analysis methods to derive quantitative parameters that are linked to the size and concentration of acoustic scatterers, as well as the texture analysis methods that quantify spatial variations and heterogeneity within the QUS parametric maps. Despite promising results in various tissue characterization applications^22–28^, QUS remains largely confined to research settings. This is because most clinical scanners do not provide access to the RF data or discard them at early processing stages to mitigate computational overhead and storage space. In this study, a solution was proposed to address this barrier by inverting the RF to B-mode conversion process using data-driven conditional generative adversarial networks. Three architectures including Pix2Pix cGAN, a shallow ViT cGAN, and a deep ViT cGAN were adapted and evaluated to synthesize realistic RF signals directly from conventional B-mode images, thereby enabling downstream QUS analysis and benign-malignant lesion characterization without requiring to store RF data. Importantly, the motivation for RF data synthesis is not simply to increase data availability, but to enable widespread access to quantitative physically interpretable QUS biomarkers that enhance lesion characterization and clinical decision-making beyond conventional B-mode ultrasound.

The results demonstrated that deep learning-based generative models can effectively synthesize RF data from B-mode images, leading to QUS features that closely approximate those extracted from the original counterparts. We evaluated the quality of the generated RF data using direct sample-level comparison metrics, qualitative comparisons of the corresponding B-mode images and QUS parametric maps, and a downstream assessment via QUS-feature-based breast lesion classification. At the sample level, all the three cGAN variants demonstrated comparable results with slight differences in performance. The models achieved a SSIM score of 0.82 ± 0.05 on the synthetic RF data, and a PSNR mean value of about 33 dB on the associated B-mode images. The Deep ViT cGAN showed the best overall performance with a NRMSE of 2.8 ± 0.6% on the synthetic RF data, and an SSIM of 0.89 ± 0.02 on the corresponding B-mode images. Qualitative comparison of the B-mode images generated from the synthetic RF data indicated that the models preserved the lesion morphology and speckle pattern of the original data. Beyond sample-wise similarity, the fidelity of synthetic RF data was further assessed through QUS analysis. UMAP analysis demonstrated a good alignment between the overall distribution patterns of the original and synthetic QUS features, particularly for the Deep ViT cGAN. Histogram analysis further confirmed that the distributions of individual QUS mean-value features were well preserved in synthetic data, particularly with respect to the central tendency and overall spread.

The utility of synthetic RF data was also validated in a downstream benign versus malignant classification task using the QUS mean and texture features, following a two-step feature selection process. Using QUS features derived from the original RF data, the SVC classifier achieved a test accuracy of 82 ± 6%. Training and testing the classifier exclusively on the synthetic data, both Deep ViT and Pix2Pix cGANs resulted in competitive performance with a test accuracy of 81 ± 6–7%., whereas the Shallow ViT cGAN lagged slightly behind (accuracy = 79 ± 6%). When the classifier was trained on the original QUS features and tested on the synthetic features, the Deep ViT cGAN maintained a comparable performance (accuracy = 84 ± 6%), outperforming both the Pix2Pix and Shallow ViT models. Finally, in experiments where the original feature subset was used for classifier training and testing with the synthetic data, the Deep ViT cGAN achieved performance matching that of the original QUS data (accuracy = 82 ± 6%), while the other models demonstrated slightly lower performance.

As an adversarial framework, the proposed models include an adversarial loss, which enables the generator and discriminator to be trained jointly in a competitive manner. While the adversarial loss is particularly important in generative tasks that emphasize novelty of the synthesized output, in paired translation problems its primary role is to encourage the synthesized outputs to follow the overall distribution and statistical characteristics of the target domain. In this study, adversarial supervision helps to ensure that the generated RF signals exhibit signal statistics consistent with the original RF data. In contrast, reconstruction losses play a more important role in paired translation tasks, where strict input-output correspondence is required. Accordingly, the L1 and L2 reconstruction losses were incorporated to minimize sample-level differences between the synthesized and ground-truth RF signals. The L1 loss emphasizes accurate reconstruction of local amplitude variations and sharp transitions, and is less sensitive to outliers, whereas the L2 loss penalizes larger deviations more strongly and contributes to training stability. Since preserving RF amplitude fidelity was our initial target, the L1 loss was assigned a higher weight than the L2 loss in the proposed framework. It is worth noting that multiple loss weight combinations were evaluated during preliminary experiments and subsequently fine-tuned based on the validation set to improve RF reconstruction quality.

From an information theory perspective, recovering RF data from B-mode images is inherently an ill-posed problem since B-mode images represent a lossy compression of RF signals. However, when a data-driven cGAN is trained on a large set of paired RF/B-mode data, it can theoretically learn a statistical model of the conditional distribution P(RF∣B-mode)^39^ for transforming B-mode images to RF data. In other words, during the training phase, the generator learns the relations between recurrent patterns like speckle textures, edges, and intensity variations in B-mode images and the high-frequency components in the RF signal. The learned correlations and statistical regularities provide rich prior information to the generative model for reconstructing plausible RF details, potentially compensating for the information loss during B-mode image generation. In this sense, the cGAN infers the lost information by exploiting the prior knowledge captured during the training process^58^, potentially yielding a high-fidelity inverse mapping from B-mode image back to RF data. The findings of this study align with those of other studies, where similar data-driven generative models have demonstrated the ability to inter-translate various medical imaging modalities with decent fidelity^33,34,37,38,59^.

This study was the first to explore the feasibility of synthesizing raw RF data from conventional B-mode images. While the results are promising and establish a proof of concept with applicability to QUS tissue characterization, the experiments were confined to a single scanner with fixed system settings. Further work should validate the proposed methods on datasets acquired using multiple ultrasound platforms with various acquisition parameters to ensure robustness and generalizability. Additionally, while the study here focused on breast lesion characterization, future studies can broaden the scope to other clinical applications, such as therapy response monitoring^16,26^ and characterization of other tissues^6^, to assess the utility of synthetic RF data across the spectrum of QUS applications.

The proposed framework relies on paired B-mode and RF data during the training or scanner-specific fine-tuning phase, which may pose a practical consideration, given that many commercial ultrasound systems do not routinely provide access to raw RF signals. However, it should be noted that access to RF data in this methodology is required only during a one-time offline training or fine-tuning stage, rather than during routine clinical deployment. Further, the limited clinical adoption of QUS imaging is not solely attributable to restricted RF data availability, but also to limitations in continuous RF data storage, transmission, and real-time processing due to the large data volume associated with RF signals. By learning a transformation from standard B-mode images to RF data during training, the proposed approach enables QUS analysis at inference time using only conventional B-mode images, thereby eliminating the need for RF data access and storage in routine clinical workflows. To obtain training data, data acquisition (DAQ) systems could potentially be adapted and integrated with a single instance of each commercial scanner model to record scanner-specific RF data, without requiring routine access to RF data on all scanners. To further facilitate clinical translation, future work may also explore scanner-specific fine-tuning strategies using limited or publicly available RF datasets, as well as training on data acquired from multiple ultrasound systems to improve robustness and generalizability and promote device-agnostic performance.

This study focused on reconstructing RF data as an intermediate representation for QUS imaging. The promising results provide a foundation for future work exploring the feasibility of directly synthesizing QUS parametric maps from conventional B-mode images using end-to-end deep-learning models. Such an approach could potentially reduce cumulative error propagation by bypassing explicit RF data reconstruction and subsequent analytical QUS estimation. However, such translation is inherently more challenging, as it requires learning multiple complex transformations within a network, which may require more data for effective model training. In addition, since QUS parameters are physically defined through frequency domain analysis of RF signals, direct end-to-end synthesis may compromise physical interpretability.

## Data Availability

Data were collected at Sunnybrook Health Sciences Centre in Toronto, ON, Canada, and are stored in an institutional repository. Data may be made available upon reasonable request, subject to review and approval by the institutional Research Ethics Board.

## References

[1] Global cancer burden growing. amidst mounting need for services, (n.d.). https://www.who.int/news/item/01-02-2024-global-cancer-burden-growing--amidst-mounting-need-for-services Accessed June 25, 2024. PMC11115397 38438207

[2] Loughran, C. F. & Keeling, C. R. Seeding of tumour cells following breast biopsy: a literature review. *Br. J. Radiol.* **84**, 869–874. 10.1259/bjr/77245199 (2011). 21933978 10.1259/bjr/77245199 PMC3473763

[3] Zhi, H. et al. Comparison of Ultrasound Elastography, Mammography, and Sonography in the Diagnosis of Solid Breast Lesions. *J. Ultrasound Med.* **26**, 807–815. 10.7863/jum.2007.26.6.807 (2007). 17526612 10.7863/jum.2007.26.6.807

[4] Ohashi, A. et al. Comparison of Ultrafast Dynamic Contrast-Enhanced (DCE) MRI with Conventional DCE MRI in the Morphological Assessment of Malignant Breast Lesions. *Diagnostics* **13**, 1105. 10.3390/diagnostics13061105 (2023). 36980417 10.3390/diagnostics13061105 PMC10046990

[5] Feleppa, E. J., Mamou, J., Porter, C. R. & Machi, J. Quantitative Ultrasound in Cancer Imaging. *Semin Oncol.* **38**, 136–150. 10.1053/j.seminoncol.2010.11.006 (2011). 21362522 10.1053/j.seminoncol.2010.11.006 PMC3057450

[6] Mamou, J. & Oelze, M. L. *Quantitative Ultrasound in Soft Tissues* (Springer, 2023).

[7] Lizzi, F. L., Greenebaum, M., Feleppa, E. J., Elbaum, M. & Coleman, D. J. Theoretical framework for spectrum analysis in ultrasonic tissue characterization. *J. Acoust. Soc. Am.* **73**, 1366–1373. 10.1121/1.389241 (1983). 6853848 10.1121/1.389241

[8] Laugier, P. & Haïat, G. (eds) *Bone Quantitative Ultrasound* (Springer Netherlands, 2011). 10.1007/978-94-007-0017-8

[9] Glüer, C. & Group, I. Q. U. C. Quantitative ultrasound techniques for the assessment of osteoporosis: expert agreement on current status. *J. Bone Miner. Res.* **12**, 1280–1288 (1997). 9258759 10.1359/jbmr.1997.12.8.1280

[10] Rocca, A. et al. Quantitative ultrasound (QUS) in the evaluation of liver steatosis: data reliability in different respiratory phases and body positions. *Radiol. Med.* **129**, 549. 10.1007/S11547-024-01786-Y (2024). 38512608 10.1007/s11547-024-01786-y PMC11021279

[11] Sharma, D. et al. Quantitative ultrasound characterization of therapy response in prostate cancer in vivo. *Am. J. Transl Res.* **13**, 4437 (2021). 34150025 PMC8205668

[12] Rohrbach, D., Wodlinger, B., Wen, J., Mamou, J. & Feleppa, E. High-Frequency Quantitative Ultrasound for Imaging Prostate Cancer Using a Novel Micro-Ultrasound Scanner. *Ultrasound Med. Biol.* **44**, 1341–1354. 10.1016/J.ULTRASMEDBIO.2018.02.014 (2018). 29627083 10.1016/j.ultrasmedbio.2018.02.014

[13] Goundan, P. N. et al. A Preliminary Study of Quantitative Ultrasound for Cancer-Risk Assessment of Thyroid Nodules. *Front. Endocrinol. (Lausanne)*. **12**, 627–698. 10.3389/FENDO.2021.627698/BIBTEX (2021). 10.3389/fendo.2021.627698 PMC8170470 34093429

[14] Sadeghi-Naini, A. et al. Low-frequency quantitative ultrasound imaging of cell death in vivo. *Med. Phys.* **40**, 082901. 10.1118/1.4812683 (2013). 23927356 10.1118/1.4812683

[15] Sadeghi-Naini, A. et al. Conventional Frequency Ultrasonic Biomarkers of Cancer Treatment Response In Vivo. *Transl Oncol.* **6**, 234–IN2. 10.1593/tlo.12385 (2013). 23761215 10.1593/tlo.12385 PMC3678128

[16] Sadeghi-Naini, A. et al. Quantitative Ultrasound Evaluation of Tumor Cell Death Response in Locally Advanced Breast Cancer Patients Receiving Chemotherapy. *Clin. Cancer Res.* **19**, 2163–2174. 10.1158/1078-0432.CCR-12-2965 (2013). 23426278 10.1158/1078-0432.CCR-12-2965

[17] Sannachi, L. et al. Non-invasive evaluation of breast cancer response to chemotherapy using quantitative ultrasonic backscatter parameters. *Med. Image Anal.* **20**, 224–236 (2015). 25534283 10.1016/j.media.2014.11.009

[18] Noritomi, T. et al. Carotid plaque typing by multiple-parameter ultrasonic tissue characterization. *Ultrasound Med. Biol.* **23**, 643–650 (1997). 9253812 10.1016/s0301-5629(97)00013-6

[19] Hoerig, C., Wallace, K., Wu, M. & Mamou, J. Classification of Metastatic Lymph Nodes In Vivo Using Quantitative Ultrasound at Clinical Frequencies. *Ultrasound Med. Biol.* **49**, 787–801. 10.1016/J.ULTRASMEDBIO.2022.10.018 (2023). 36470739 10.1016/j.ultrasmedbio.2022.10.018

[20] Oelze, M. L., O’Brien, W. D., Blue, J. P. & Zachary, J. F. Differentiation and characterization of rat mammary fibroadenomas and 4T1 mouse carcinomas using quantitative ultrasound imaging. *IEEE Trans. Med. Imaging*. **23**, 764–771 (2004). 15191150 10.1109/tmi.2004.826953

[21] Oelze, M. L. & Zachary, J. F. Examination of cancer in mouse models using high-frequency quantitative ultrasound. *Ultrasound Med. Biol.* **32**, 1639–1648. 10.1016/j.ultrasmedbio.2006.05.006 (2006). 17112950 10.1016/j.ultrasmedbio.2006.05.006

[22] Liao, Y. et al. Classification of scattering media within benign and malignant breast tumors based on ultrasound texture-feature‐based and Nakagami‐parameter images. *Med. Phys.* **38**, 2198–2207. 10.1118/1.3566064 (2011). 21626954 10.1118/1.3566064

[23] Gomez, W., Pereira, W. C. A. & Infantosi, A. F. C. Analysis of Co-Occurrence Texture Statistics as a Function of Gray-Level Quantization for Classifying Breast Ultrasound. *IEEE Trans. Med. Imaging*. **31**, 1889–1899. 10.1109/TMI.2012.2206398 (2012). 22759441 10.1109/TMI.2012.2206398

[24] Sadeghi-Naini, A. et al. Breast-Lesion Characterization using Textural Features of Quantitative Ultrasound Parametric Maps. *Sci. Rep.* **7**, 7963. 10.1038/s41598-017-13977-x (2017). 10.1038/s41598-017-13977-x PMC5651882 29057899

[25] Osapoetra, L. O. et al. Breast lesion characterization using Quantitative Ultrasound (QUS) and derivative texture methods. *Transl Oncol.* **13 (10)**, 100827. 10.1016/j.tranon.2020.100827 (2020). 10.1016/j.tranon.2020.100827 PMC7358267 32663657

[26] Sadeghi-Naini, A. et al. Chemotherapy-Response Monitoring of Breast Cancer Patients Using Quantitative Ultrasound-Based Intra-Tumour Heterogeneities. *Sci. Rep.* **7**, 10352. 10.1038/s41598-017-09678-0 (2017). 10.1038/s41598-017-09678-0 PMC5583340 28871171

[27] Sadeghi-Naini, A. et al. Early prediction of therapy responses and outcomes in breast cancer patients using quantitative ultrasound spectral texture. *Oncotarget* **5**, 3497 (2014). 24939867 10.18632/oncotarget.1950 PMC4116498

[28] Taleghamar, H., Moghadas-Dastjerdi, H., Czarnota, G. J. & Sadeghi-Naini, A. Characterizing intra-tumor regions on quantitative ultrasound parametric images to predict breast cancer response to chemotherapy at pre-treatment. *Sci. Rep.* **11**, 14865. 10.1038/s41598-021-94004-y (2021). 10.1038/s41598-021-94004-y PMC8295369 34290259

[29] Taleghamar, H., Jalalifar, S. A., Czarnota, G. J. & Sadeghi-Naini, A. Deep learning of quantitative ultrasound multi-parametric images at pre-treatment to predict breast cancer response to chemotherapy. *Sci. Rep.* **12**, 2244. 10.1038/s41598-022-06100-2 (2022). 35145158 10.1038/s41598-022-06100-2 PMC8831592

[30] Goodfellow, I. J. et al. Generative Adversarial Networks. in *Proceedings of the 27th International Conference on Neural Information Processing Systems* **2**, 2672-2680. https://dl.acm.org/doi/10.5555/2969033.2969125 (2014).

[31] Kingma, D. P. & Welling, M. An Introduction to Variational Autoencoders. *Found. Trends Mach. Learn.* **12****(4)**, 307–392. 10.1561/2200000056 (2019).

[32] Ronneberger, O., Fischer, P. & Brox, T. U-net: Convolutional networks for biomedical image segmentation. in *Medical Image Computing and Computer-Assisted Intervention–MICCAI 2015: 18th International Conference, Munich, Germany, October 5–9, 2015, Proceedings, Part III 18* 234–241 (Springer, 2015). 10.1007/978-3-319-24574-4_28

[33] Yang, Q. et al. MRI cross-modality image-to-image translation. *Sci. Rep.* **10**, 3753 (2020). 32111966 10.1038/s41598-020-60520-6 PMC7048849

[34] Wang, T. et al. A review on medical imaging synthesis using deep learning and its clinical applications. *J. Appl. Clin. Med. Phys.* **22**, 11–36. 10.1002/acm2.13121 (2021). 33305538 10.1002/acm2.13121 PMC7856512

[35] Bahrami, K. et al. Reconstruction of 7T-Like Images From 3T MRI. *IEEE Trans. Med. Imaging*. **35**, 2085–2097. 10.1109/TMI.2016.2549918 (2016). 27046894 10.1109/TMI.2016.2549918 PMC5147737

[36] Becker, M. et al. Deep learning corrects artifacts in RASER MRI profiles. *Magn. Reson. Imaging*. **115**, 110247. 10.1016/j.mri.2024.110247 (2025). 39461486 10.1016/j.mri.2024.110247

[37] Shin, H. C. et al. Medical Image Synthesis for Data Augmentation and Anonymization Using Generative Adversarial Networks. in *Proceedings of Third International Workshop on Simulation and Synthesis in Medical Imaging* 1–11 (2018). 10.1007/978-3-030-00536-8_1

[38] Kebaili, A., Lapuyade-Lahorgue, J. & Ruan, S. Deep Learning Approaches for Data Augmentation in Medical Imaging: A Review. *J. Imaging*. **9**, 81. 10.3390/jimaging9040081 (2023). 37103232 10.3390/jimaging9040081 PMC10144738

[39] Isola, P., Zhu, J. Y., Zhou, T. & Efros, A. A. Image-to-image translation with conditional adversarial networks. in *Proceedings of the IEEE Conference on Computer Vision and Pattern Recognition* 1125–1134 (2017). https://doi.ieeecomputersociety.org/10.1109/CVPR.2017.632

[40] Zhu, J. Y., Park, T., Isola, P. & Efros, A. A. Unpaired Image-to-Image Translation using Cycle-Consistent Adversarial Networks. in *Proceedings of the IEEE International Conference on Computer Vision* 2242-2251 (2017). 10.1109/ICCV.2017.244

[41] Dalmaz, O., Yurt, M. & Cukur, T. ResViT: Residual Vision Transformers for Multimodal Medical Image Synthesis. *IEEE Trans. Med. Imaging*. **41**, 2598–2614. 10.1109/TMI.2022.3167808 (2022). 35436184 10.1109/TMI.2022.3167808

[42] Kamran, S. A., Hossain, K. F., Tavakkoli, A., Zuckerbrod, S. L. & Baker, S. A. VTGAN: Semi-supervised Retinal Image Synthesis and Disease Prediction using Vision Transformers. in *Proceedings of the IEEE/CVF International Conference on Computer Vision Workshops* 3235-3245 (2021). 10.1109/ICCVW54120.2021.00362

[43] Wang, X. et al. Conditional Diffusion Model for Abdominal CT Image Synthesis. in *22nd International Symposium on Biomedical Imaging (ISBI)* 1–5 (IEEE, 2025). 10.1109/ISBI60581.2025.10980773

[44] Müller-Franzes, G. et al. A multimodal comparison of latent denoising diffusion probabilistic models and generative adversarial networks for medical image synthesis. *Sci. Rep.* **13**, 12098. 10.1038/s41598-023-39278-0 (2023). 37495660 10.1038/s41598-023-39278-0 PMC10372018

[45] Rangarajan, K. et al. Simulation training in mammography with AI-generated images: a multireader study. *Eur. Radiol.* **35**, 562–571. 10.1007/s00330-024-11005-x (2024). 39134745 10.1007/s00330-024-11005-x

[46] Ren, J. *From RF signals to B-mode images using deep learning, Master’s degree* (KTH Royal Institute of Technology, 2018).

[47] Rai, H. M., Dashkevych, S., Yoo, J. & Diagnostics, N. G. The Impact of Synthetic Data Generation on the Detection of Breast Cancer from Ultrasound Imaging. *Mathematics* **12**, 2808. 10.3390/math12182808 (2024).

[48] Oelze, M. L. & O’Brien, W. D. Frequency-dependent attenuation-compensation functions for ultrasonic signals backscattered from random media. *J. Acoust. Soc. Am.* **111**, 2308–2319. 10.1121/1.1452743 (2002). 12051451 10.1121/1.1452743

[49] Labyed, Y. & Bigelow, T. A. Estimating the total ultrasound attenuation along the propagation path by using a reference phantom. *J. Acoust. Soc. Am.* **128**, 3232–3238. 10.1121/1.3483739 (2010). 21110618 10.1121/1.3483739 PMC3003735

[50] Berger, G., Laugier, P., Thalabard, J. C. & Perrin, J. Global Breast Attenuation: Control Group and Benign Breast Diseases. *Ultrason. Imaging*. **12**, 47–57. 10.1177/016173469001200104 (1990). 2184568 10.1177/016173469001200104

[51] Duric, N. et al. Development of ultrasound tomography for breast imaging: Technical assessment. *Med. Phys.* **32**, 1375–1386. 10.1118/1.1897463 (2005). 15984689 10.1118/1.1897463

[52] Tadayyon, H. et al. Quantitative ultrasound assessment of breast tumor response to chemotherapy using a multi-parameter approach. *Oncotarget* **7**, 45094–45111. 10.18632/oncotarget.8862 (2016). 27105515 10.18632/oncotarget.8862 PMC5216708

[53] Tadayyon, H., Sadeghi-Naini, A., Wirtzfeld, L., Wright, F. C. & Czarnota, G. Quantitative ultrasound characterization of locally advanced breast cancer by estimation of its scatterer properties. *Med. Phys.* **41**, 12903–12904. 10.1118/1.4852875 (2014). 10.1118/1.4852875 24387530

[54] Haralick, R. M., Shanmugam, K. & Dinstein, I. Textural Features for Image Classification. *IEEE Trans. Syst. Man. Cybern SMC*. **-3**, 610–621. 10.1109/TSMC.1973.4309314 (1973).

[55] Pedregosa, F. et al. Scikit-learn: Machine Learning in Python. *J. Mach. Learn. Res.* **12** 2825-2830 (2011).

[56] McInnes, L., Healy, J., Saul, N. & Großberger, L. Uniform Manifold Approximation and Projection. *J. Open. Source Softw.* **3**, 861. 10.21105/joss.00861 (2018).

[57] Zou, H. & Hastie, T. Regularization and Variable Selection Via the Elastic Net. *J. R Stat. Soc. Ser. B Stat. Methodol.* **67**, 301–320. 10.1111/j.1467-9868.2005.00503.x (2005).

[58] Dong, C., Loy, C. C., He, K. & Tang, X. Image Super-Resolution Using Deep Convolutional Networks. *IEEE Trans. Pattern Anal. Mach. Intell.* **38****(2)**, 295-307. 10.1109/TPAMI.2015.2439281 (2016). 10.1109/TPAMI.2015.2439281 26761735

[59] Yang, Q. et al. MRI cross-modality neuroimage-to-neuroimage translation. *Sci. Rep.* **10**, 3753. 10.1038/s41598-020-60520-6 (2018). 10.1038/s41598-020-60520-6 PMC7048849 32111966

